# Functional structure and antimicrobial activity of persulcatusin, an antimicrobial peptide from the hard tick *Ixodes persulcatus*

**DOI:** 10.1186/s13071-016-1360-5

**Published:** 2016-02-13

**Authors:** Naruhide Miyoshi, Takeshi Saito, Tadahiro Ohmura, Kengo Kuroda, Kazumasa Suita, Kohei Ihara, Emiko Isogai

**Affiliations:** Department of Animal Microbiology, Graduate School of Agricultural Science, Tohoku University, 1-1 Tsutsumidori Amamiya-machi, Aoba-ku, Sendai, Miyagi 981-8555 Japan; Dassault Systemes Biovia K.K, Shinagawa-ku, Tokyo Japan

**Keywords:** Tick, Antimicrobial peptide, Persulcatusin, S-S bond, Methicillin-resistant Staphylococcus aureus

## Abstract

**Background:**

Antimicrobial peptides (AMPs) are considered promising candidates for the development of novel anti-infective agents. In arthropods such as ticks, AMPs form the first line of defense against pathogens in the innate immune response. Persulcatusin (IP) was found in the *Ixodes persulcatus* midgut, and its amino acid sequence was reported. However, the complete structure of IP has not been identified. We evaluated the relation between structural features and antimicrobial activity of IP, and its potential as a new anti-methicillin-resistant *Staphylococcus aureus* (MRSA) agent.

**Methods:**

The structure of IP was predicted using homology modeling and molecular dynamics. IP and other tick AMPs were synthesized using a solid-phase method and purified by high-performance liquid chromatography. Methicillin-susceptible *S. aureus* (MSSA) and MRSA were used for the minimum inhibitory concentration (MIC) test and short-time killing assay of IP and other tick peptides. The influence of IP on mammalian fibroblasts and colon epithelial cells and each cell DNA and its hemolytic activity towards human erythrocytes were also examined.

**Results:**

In the predicted IP structure, the structure with an S-S bond was more stable than that without an S-S bond. The MIC after 24 h of incubation with IP was 0.156–1.25 μg/mL for MSSA and 0.625–2.5 μg/mL for MRSA. Compared with the mammalian antimicrobial peptide and other tick peptides, IP was highly effective against MRSA. Moreover, IP showed a dose-dependent bactericidal effect on both MSSA and MRSA after 1 h of incubation. IP had no observable effect on mammalian cell growth or morphology, on each cell DNA and on human erythrocytes.

**Conclusions:**

We predicted the three-dimensional structure of IP and found that the structural integrity was maintained by three S-S bonds, which were energetically important for the stability and for forming α helix and β sheet. IP has cationic and amphipathic properties, which might be related to its antimicrobial activity. Furthermore, the antimicrobial activity of IP against MRSA was stronger than that of other antimicrobial peptides without apparent damage to mammalian and human cells, demonstrating its possible application as a new anti-MRSA medicine.

## Background

Multidrug-resistant bacteria are a severe threat to public health. Conventional antibiotics are becoming increasingly ineffective because of such resistance, and it is imperative to find new antibacterial strategies [1]. Antimicrobial peptides (AMPs) are an integral part of the innate immune system of all living organisms and are considered promising candidates for the development of novel anti-infective agents [2]. These molecules have a broad antimicrobial activity spectrum, various modes of action, and decreased incidence of resistance development [3, 4]. A major AMP family is the defensin family found in various organisms including plants, vertebrates, and invertebrates [5]. AMPs of arthropods, who have a powerful innate immune response, are included in this family [6].

Ticks are external hematophagous parasites that live on the blood of mammals, birds, and, occasionally, reptiles and amphibians. A handful of ticks are vectors of many diseases that affect both humans and other animals [7]. *Ixodes persulcatus* is a predominant tick species that spreads a wide array of serious human and animal pathogens, including *Borrelia garinii*, which causes Lyme disease. In Japan, Lyme disease in humans is due to infection with *B. garinii* or *B. afzelii*, which are specifically transmitted by *I. persulcatus* [8]. Despite the ability of ticks to harbor and transmit pathogens, their immune system offers effective mechanisms against pathogenic microorganisms in the event of their permeation into the tick body [9].

In ticks, AMPs form the first line of defense against pathogens in the innate immune response [10]. Tick AMPs have been detected in several tissues, such as the midgut and salivary glands, and can be inoculated into host bodies during blood meals [11, 12]. Persulcatusin (IP), a tick AMP in the *I. persulcatus*, was found in the tick midgut and its amino acid sequence was reported [13]. Furthermore, this AMP has antimicrobial activity against gram-positive bacteria such as *Staphylococcus aureus* [13]. Most AMPs from insects and arthropods conserve a characteristic motif of six cysteines, which form three disulfide bonds [14, 15]. Tick AMPs are well known and the most widely characterized among antimicrobial molecules [9, 11, 12, 16, 17]. Similar to other tick AMPs, IP contains six cysteine residues that may form S-S bonds, but the structure of IP has not been identified. Our research group previously investigated the relation between the antimicrobial activity of IP and its three-dimensional and primary structure, and found that IP with a three-dimensional structure was more effective to gram-positive bacteria compared to that with a primary structure [18]. Therefore, to prove the importance of the three-dimensional structure of tick, it is important to perform prediction of IP structure.

Hundreds of AMPs have been isolated and characterized to understand their mode of action, but many AMPs have limited use as therapeutics, owing to their cytotoxicity against mammalian cells [19–21]. IP may have the same problem. However, the first symptom of Lyme disease is reported to be an erythema migrans, a type of inflammation of the skin [22], which is affected by molecules present in tick saliva [23]. The candidate molecules that cause this inflammation include the tick AMP because AMPs have an immunomodulation ability [24]. To examine these effects, the ability of IP to affect mammalian cells and its hemolytic activity against human erythrocytes was tested.

*I. persulcatus* have feeding activities. Our study group found that *S. aureus* cannot be isolated from *I. persulcatus* during feeding [16]. This is thought to be caused by the antimicrobial activity of IP [13, 18]. *S. aureus* is potentially pathogenic and can adapt rapidly to the selective pressure of antibiotics [25]. In particular, methicillin-resistant *S. aureus* (MRSA) infections have become major public health concern. Since the late 1990s, community-associated MRSA has emerged as a principal cause of skin and soft-tissue epidemics worldwide [26, 27]. As the *Journal of the American Medical Association* reported in 2007, there were an estimated 94,360 cases of MRSA infections in the United States in 2005 [28].

In this study, we predicted the three-dimensional structure of IP by homology modeling and synthesized IP and other tick AMPs to evaluate their antimicrobial activity against MRSA. In addition, we examined the toxicity of IP toward mammalian cells. Throughout this study, we evaluated the relation between structural feature and antimicrobial activity of IP, and the potential of IP as a new anti-MRSA agent.

## Methods

### Homology modeling

The amino acid sequence of tick AMP IP was GFGCPFNQGACHRHCRSIGRRGGYCAGLFKQTCTCYSR (AB469201). The template structure was selected by searching PubMed for structures including >1 α helix and >1 β sheet. We superposed the structures and classified clusters for the template-structure candidates by using three-dimensional structure multiple alignments. We assumed each cluster to be a template. Sequences were aligned by manual correction using the results of three-dimensional structure multiple alignments. We produced five structures by the Build Homology Model protocol of Discovery Studio® 2.5 (Dassault Systèmes BIOVIA, San Diego) and selected one homology model from each template structure by applying the following standards sequentially;

1. Probability density function (PDF) Total Energy, PDF Physical Energy, and Discrete optimized protein energy (DOPE) Score was superior to that of other structures
2. There were fewer residues outside the domain compared to other structures in the Ramachandran Plot.
3. High rank of the Verify Score provided in Verify Protein (Profile-3D)

From these homology models, we built each structure with or without an SS combination and optimized hydrogen atoms by using the Chemistry at HARvard Macromolecular Mechanics (CHARMm) force field. We used the Minimization protocol for structure optimization.

### Molecular dynamics

For each initial structure obtained by homology modeling, we performed a fixed temperature simulation of the Generalized Born with a simple SWitching (GBSW) Implicit Solvent Model in 10 nsec at 300 K by using the Standard Dynamics Cascade and sampled the structure every 1 psec. The representative structure among the sampled structure was selected using the following protocol:

1. Sort sampling structures by using a Root mean square deviation (RMSD) score from the initial structure, distribute it into 1000 parts, and build 10 structure ensembles
2. Calculate the intersection RMSD in each group and carry out segmented hierarchical clustering by using the distance matrix
3. Select the representative structure by using a threshold based on a dendrogram of the clustering
4. Optimize by energy minimization

In addition, we selected the structure with the lowest potential energy in each cluster, minimized its energy, and estimated the final structure. All these calculations were performed using Discovery Studio 2.5®.

### Peptide synthesis and purification

Tick and mammalian peptides were synthesized by the solid-phase method, as previously described [16]. The peptides were purified by reverse-phase high-performance liquid chromatography (Model LC-8A; Shimadzu Corporation, Kyoto, Japan) on a YMC-A 302 column. The final products were confirmed by electrospray ionization mass spectrometry and were supplied as trifluoroacetates. This trifluoroacetate form of the peptides was conserved by suspending in Hanks’ Balanced Salt Solution (HBSS; GIBCO, Grand Island, NY, USA) at pH 7.4 and stored at −20 °C. IR, HAE, and OMBAC were the tick AMPs, and for mammalian AMP, a bovine myeloid antimicrobial peptide (BMAP28) was used. Their sequences have been reported [11, 29–31].

### Bacterial strain and culture conditions

In the growth inhibition test, we used 9 clinical strains of methicillin-susceptible *S. aureus* (MSSA) and 9 clinical strains of MRSA from patients in Jichi Medical University Hospital, using these strains in past report [31]. The bacteria were grown in Trypto Soya (TS; Nissui, Tokyo, Japan) broth for 18–19 h at 37 °C.

### Growth inhibition test

The optical density at 660 nm (OD_660_) of pre-cultured bacteria was measured using an Ubest-35 (JASCO Corporation, Tokyo, Japan). The adjustment for an OD_660_ of 0.5 was conducted by adding TS broth. The bacteria were diluted to a final concentration of 1–5 × 10^4^ colony forming units (CFUs) /mL with TS broth, after which 50 μL of bacterial suspension and 50 μL of peptide solution were mixed together in a 96-well plate. The peptide solution was prepared by two-fold dilution in TS broth, while the IP solution was prepared to final peptide concentrations of 40, 20, 10, 5, 2.5, 1.25, 0.625, 0.313, 0.157, and 0.079 μg/mL. Each mixture of bacteria and peptide solution was incubated at 37 °C. The OD_660_ of the cell suspension was measured after 20–24 h incubation by using a Synergy™ HT (BioTek, Winooski, VT, USA). A control was prepared by mixing 50 μL of bacterial suspension, 40 μL of TS broth, and 10 μL of HBSS. The minimal inhibitory concentration (MIC) of the peptides was defined as the lowest concentration of peptide that reduced growth by >90 %.

### Short-time killing assay

We selected 3 strains each of MSSA and MRSA used in the growth inhibition test. Unlike the growth inhibition test described above, an additional growth inhibition test was conducted using small test tubes. Each mixture of bacteria and peptide solution was incubated for 1 h at 37 °C. After the incubation, 100 μL aliquots were removed from the tubes and inoculated on TS agar plates. The plates were incubated overnight at 37 °C, after which colonies were counted. The same control was prepared as in the growth inhibition test described above. The data were then converted and expressed as a percentage (sample per control).

### Effect on mammalian cell growth and morphology

In this study, we used bovine fetal fibroblasts (BFFs-NCC1) and bovine fetal colon epithelial cells (BFCEs-K4DT), which were maintained in the Laboratory of Animal Breeding and Genetics, Tohoku University Graduate School of Agricultural Science [32, 33]. The BFFs-NCC1 and BFCEs-K4DT were cultured in a cell culture dish and wrapped 12-well plates. The nutrient medium of each cell was Dulbecco’s modified Eagle’s medium (DMEM; Nacalai Tesque, Kyoto, Japan or Mediatech, Inc, Manassas, VA, USA) containing 10 % fetal bovine serum (Invitrogen, Carlsbad, CA, USA) and a 1 % antibiotic-antimycotic mixed stock solution (Nacalai Tesque). Then each cell was seeded into 12-well plates at 1 × 10^4^ cell/well and 1 × 10^5^ cell/well. After overnight incubation, the nutrient mediums were changed to DMEM with IP (0, 5, 10, 50 μg/mL), and cells were incubated for 24 h or 48 h (BFCEs-K4DT only 48 h) at 37 °C. Then, these were collected and measured using a trypan blue staining method, with an EVE™ Automatic cell counter (NanoEnTeK, Seoul, Korea). For BFCEs-K4DT, the morphology of untreated control (IP 0 μg/mL) and treated cell (IP 50 μg/mL) was observed.

### Neutral comet assay

We prepared BFFs-NCC1 and BFCEs-K4DT treated or untreated with IP 50 μg/mL for 48 h as above experiment. These cells were harvested by centrifugation (800 g, 3 min) and washed with sterile Dulbecco’s Phosphate Buffered Saline (DPBS; GIBCO). To generate positive control for comet tails, a part of each cell is untreated with IP treated 25 μM KMnO_4_, and all samples placed for 20 min at 4 °C. The comet assay was performed under neutral conditions following the Trevigen protocol. Each cell at 1 × 10^5^ cell/mL was combined with molten low-melting agarose at a ratio of 1 : 10 (vol /vol) and immediately pipetted 50 μL onto Comet slides. Slides were stored in the dark for 30 min at 4 °C before immersing in lysis solution overnight. The slides were immersed in freshly prepared neutral electrophoresis buffer (dissolved Tris base and sodium acetate in distilled water, pH 9.0) for 30 min at 4 °C. Gel electrophoresis was performed at 1 volt per cm (measured electrode to electrode) for 45 min in neutral electrophoresis buffer. The comet slides were immersed in DNA precipitation solution (7.5 M ammonium acetate containing 95 % ethanol) for 30 min at room temperature and then in 70 % ethanol for 30 min at room temperature. After drying for 10 min at 37 °C, a 100 μL of diluted Hoechst solution (Invitrogen) was placed onto each dried agarose circle. The slides were observed by fluorescence microscopy FSX100 (Olympus, Tokyo, Japan).

### Hemolytic assay

The hemolytic activity of tick peptides was determined using human erythrocytes. Erythrocytes were harvested by centrifugation (400 g, 10 min) and washed three times with sterile DPBS. A suspension of erythrocytes (2 %; vol/vol) was used for the assay. A stock solution of tick peptides was diluted in DPBS and co-incubated with erythrocytes for 2 h at 37 °C at a final volume of 100 μL and final concentrations of 0–200 μg/mL. After incubation, the suspension was centrifuged (400 g, 10 min); 100 μL of supernatant was removed, and the absorbance of samples was measured at 405 nm (An). The hemolytic activity was calculated in relation to negative and positive controls (% hemolysis = (An – A_0_/A_100_ – A_0_) × 100; A_0_ = 0 % hemolysis in DPBS; A_100_ = 100 % hemolysis obtained by incubation with 0.2 % solution of Triton X-100 in DPBS).

## Results

### Structural analysis of IP with homology modeling

**Fig. 1:**
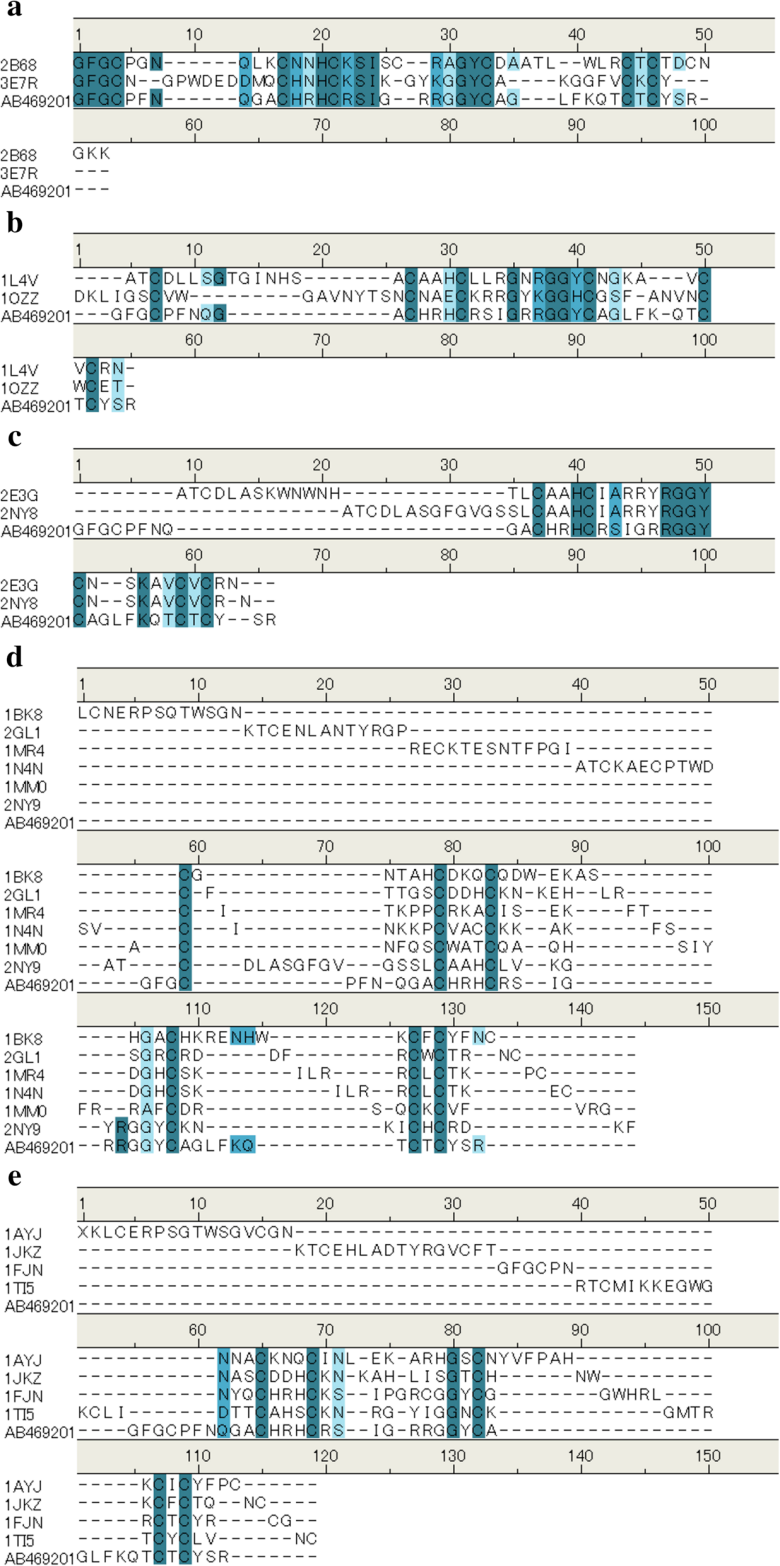
Sequence alignment of each cluster. By superposing the 20 structures with three-dimensional structure alignment and classifying cluster with the similarity, the 5 clusters were made by 16 structures. The clusters are cluster 1 (**a**), cluster 2 (**b**), cluster 3 (**c**), cluster 4 (**d**), and cluster 5 (**e**). Other 4 structures are Orphan. AB469201 was the amino acid sequence of IP and other accession numbers are described in Table 1

After obtaining 84 structures related to the keyword “Defensin” by a PubMed search, 20 structures were found to correspond because of selection of structures with only >1 α helix and >1 β sheet (Table 1). By superposing these 20 structures by three-dimensional structure alignment and classifying similar structures into clusters, 5 clusters were obtained from 16 structures. Based on a template-structure cluster and adjusted alignment (Fig. 1), we built 5 homology models for each template-structure cluster by using the Build Homology Model protocol. Moreover, as an initial structure for molecular dynamics, we built 2 structural patterns with and without 3 sets of disulfide combinations for all homology models. For each structure, we optimized the position of hydrogen atoms by energy minimization and built 10 initial structures for simulation.

**Table 1:** Selected 20 structures from Protein Data Bank (PDB)

Accession numbers	Species	Category	Method
1AYJ	*Raphanus sativus Var. niger*	Plant	NMR
1BK8	*Aesculus hippocastanum*	Plant	NMR
1JKZ	*Pisum sativum*	Plant	NMR
1MR4	*Nicotiana tabacum*	Plant	NMR
1N4N	*Petunia x hybrida*	Plant	NMR
1TI5	*Vigna radiata*	Plant	NMR
1UGL	*Brassica rapa*	Plant	NMR
2GL1	*Vigna radiata*	Plant	NMR
1L4V	*Sarcophaga peregrina*	Insect	NMR
1MM0	*Pseudacanthotermes spiniger*	Insect	NMR
1OZZ	*Archaeoprepona demophon*	Insect	NMR
2E3E	*Anopheles gambiae*	Insect	NMR
2E3F	*Anopheles gambiae*	Insect	NMR
2E3G	*Anopheles gambiae*	Insect	NMR
2NY8	*Anopheles gambiae*	Insect	NMR
2NY9	*Anopheles gambiae*	Insect	NMR
2NZ3	*Anopheles gambiae*	Insect	NMR
1FJN	*Mediterranean mussel*	Bivalve	NMR
2B68	*Crassostrea gigas*	Bivalve	NMR
3E7R	*Pseudoplectania nigrella*	Fungi	X-ray

**Fig. 2:**
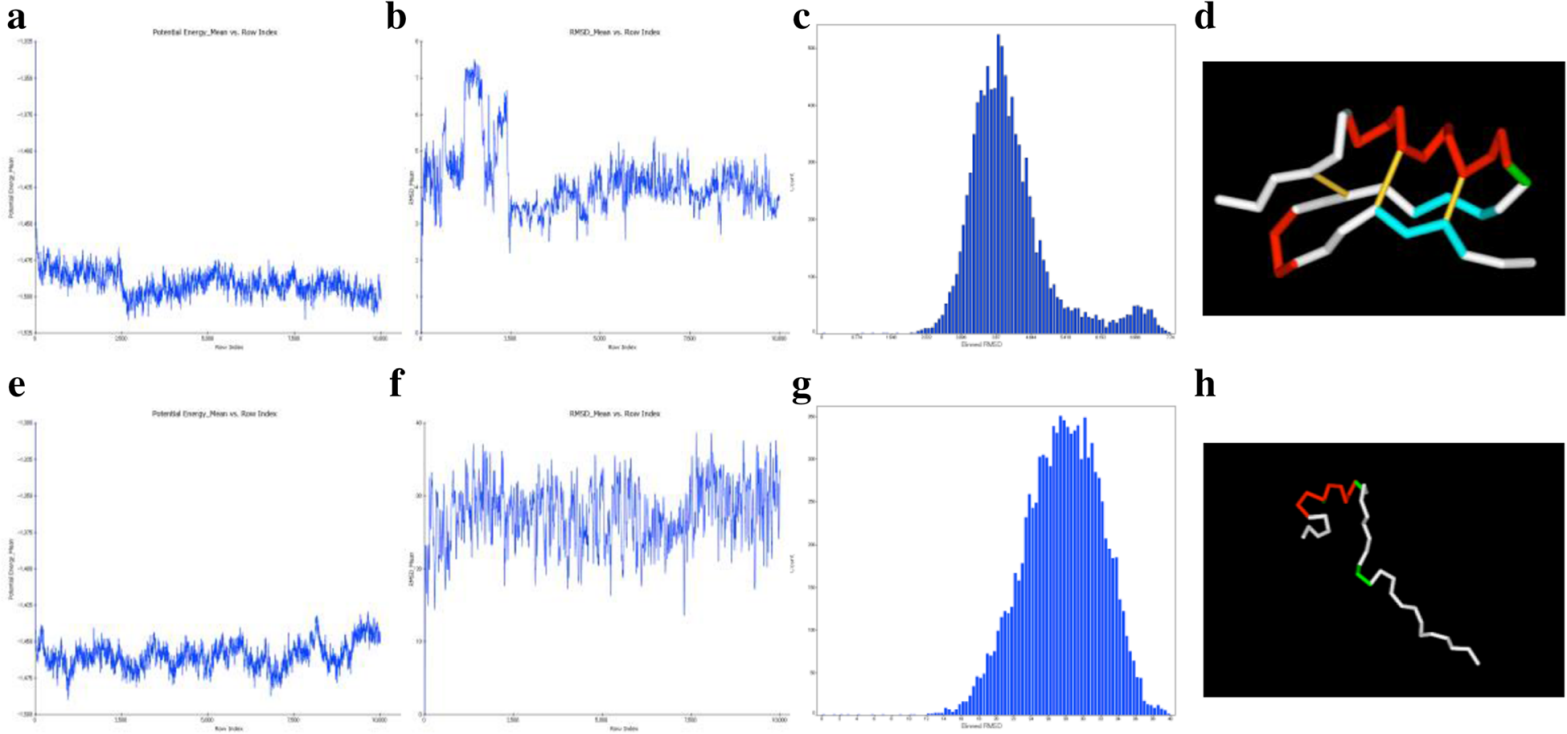
Potential energy, RMSD plot, RMSD histogram and output structure of MD simulation results. We performed Molecular dynamics with 10 structures obtained by Homology modeling. Each figure shows total potential energy (**a**, **e**), root mean square deviation (RMSD) (**b**, **f**), RMSD histogram (**c**, **g**) and output structure (**d**, **h**). In simulation with S-S bond, we select the result of Cluster-4 for stable structure group (**d**) because it is considered that the result of Cluster-4 has been lowest potential energy transition (**a**) and most stable condition. As a result of simulation without S-S bond, it is shown that RMSD score (**f**) has been remarkably high and structure (**h**) have greatly changed in the simulation process compared to simulation with S-S bond

We performed molecular dynamics analysis on the 10 structures obtained by homology modeling. Because of sampling the structures by using the Standard Dynamics Cascade, we obtained 10,000 structures for every simulation. Then from the simulation results, we obtained the changes in RMSD for the initial structure, potential energy, and number of the samples of the initial structures with and without the S-S bond (Fig. 2). In simulations with the S-S bond, we selected Cluster-4 as a stable structure group because it had the lowest potential energy transition and most stable condition (Fig. 2a). We selected a representative structure from this cluster as described above. In contrast, in simulations without the S-S bond, the RMSD score was remarkably high and the structure changed greatly in the simulation process than in the simulation with the S-S bond (Fig. 2f). Furthermore, we found that the RMSD changes greatly in a short time after simulation start and the initial structural motif was not energetically stable in simulations without S-S bond.

**Fig. 3:**
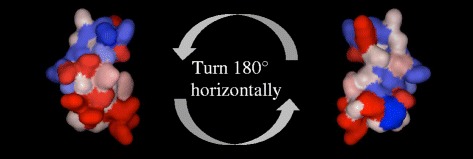
Tertiary structure and the property of IP. In the structure with S-S bond, it is confirmed that one endpoint had been hydrophilic (*red*), and other endpoint had been hydrophobic (*blue*) as a whole. Arg residues are concentrated in red domain and Phe and Leu are concentrated in blue domain

In the structure with S-S bond, among amino acid residues, Arg residues were concentrated from the end of the helix to the seat structure, forming a domain with very high hydrophilicity (Fig. 3). At the opposite end to this hydrophilicity domain, hydrophobic residues such as Phe2, Leu28, and Phe29 were concentrated, and a domain with high hydrophobicity was formed. Therefore, it was confirmed that one endpoint was hydrophilic and the other endpoint was hydrophobic (Fig. 3). On the other hand, in the structure without the S-S bond, the contrast in hydrophobic and hydrophilic domains, as seen above, was not observed, and it was not confirmed whether hydrophilic and hydrophobic residues are always concentrated in the neighborhood.

### Antimicrobial activity assay

The amino acid sequences in this study are presented in Table 2, including properties of each specific peptide. Results of the growth inhibition test showed that IP inhibited the growth of MSSA and MRSA, corresponding to MIC of 0.156–1.25 μg/mL and 0.625–2.5 μg/mL, respectively (Table 3).

**Table 2:** Amino acid sequences and properties of tick AMPs, mammal AMP

Tick species	Product name	Amino acid sequence	Similarity to IP (%)	Net charge	Hydrophobic (%)
*Ixodes persulcatus*	IP	[13] GFGCPFNQGACHRHCRSIGRRGGYCAGLFKQTCTCYSR	―	+6	39.47
*Ixodes ricinus*	IR	[29] GGYYCPFFQDKCHRHCRSFGRKAGYCGGFLKKTCICV	71	+5	45.95
*Haemaphysalis longicornis*	HAE	[30] GCPLNQGACHNHCRSIGRRGGYCAGIIKQTCTCYRK	88	+6	38.89
*Ornithodoros moubata*	OMBAC	[11] GFGCPFNQYECHAHCSGVPGYKGGYCKGLFKQTCNCY	75	+2	43.24
*Ornithodoros moubata*	(No use)	[17] GYGCPFNQYQCHSHCSGIRGYKGGYCKGTFKQTCKCY	72	+5	37.84
(Mammal AMP)	BMAP28	[31] GGLRSLGKKILRAWKKYGPIIVPIIRI		+7	48.15

Amino acid sequence was cited in each reference [11, 13, 17, 29–31]. Net charge: calculated value by charged amino acid (Lys + Arg-Asp-Glu). Hydrophobic(%): hydrophobic proportion in amino acid sequence composition by Protein/Peptide Property Calculator (http://lifetein.com/peptide-analysis-tool.html)

**Table 3:** MIC of IP against MSSA and MRSA

Bacteria strain (MSSA)	MIC (μg/mL)	Bacteria strain (MRSA)	MIC (μg/mL)
MS-1	1.25	MR-1	1.25
MS-2	1.25	MR-2	2.5
MS-3	0.156	MR-3	2.5
MS-4	0.313	MR-4	0.625
MS-5	0.313	MR-5	1.25
MS-6	0.625	MR-6	2.5
MS-7	0.313	MR-7	1.25
MS-8	0.625	MR-8	2.5
MS-9	0.313	MR-9	0.625

MS-1-9: Clinical isolates of MSSA. MR-1-9: Clinical isolates of MRSA

The antibacterial activity of synthetic tick AMPs was compared with that of other cationic antibacterial peptides (Table 4). Anti-MRSA activity of IP was stronger than that of other AMPs.

**Table 4:** Comparison of MICs against MRSA

Bacteria strain (MRSA)	MIC (μg/mL)				
	IP	IR	HAE	OMBAC	BMAP28
MR-1	1.25	20	>40	10	10
MR-2	2.5	40	>40	10	20
MR-3	2.5	10	>40	5	20

The results were confirmed by three independent experiments

### Short-time killing assay

**Fig. 4:**
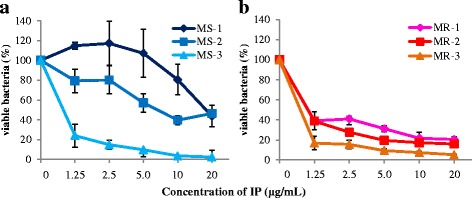
Short time killing assay. We selected each 3 strain MSSA and MRSA used in growth inhibition test. As a control, the same in the growth inhibition test was prepared. **a** In MSSA, MS-3 shows high sensitivity and MS-1 and MS-2 show low sensitivity to IP in 1 h. In both cases, IP has dose dependent bactericidal activity. **b** In MRSA, IP shows strong bactericidal activity in 1 h and dose dependent effect against all 3 strains. The means and standard deviations of triplicate determinations are presented

A short-time killing assay was employed to determine bactericidal activity of IP against MSSA and MRSA in 1 h, results of which are shown in Fig. 4. For MSSA, MS-3 was susceptible to IP and the others such as MS-1 and MS-2 were relatively resistant to IP. In contrast, all MRSA strains (MR-1, 2, 3) were susceptible to IP. Taken together, IP showed dose-dependent bactericidal activity against MSSA and MRSA within 1 h.

### Effect on mammalian cells, neutral comet assay and hemolytic assay

**Fig. 5:**
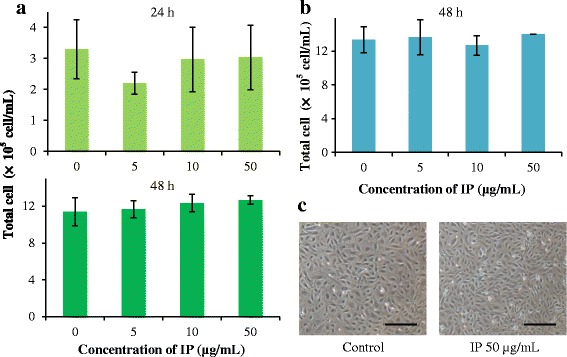
The total number and morphological images of BFFs-NCC1 and BFCEs-K4DT treated with IP in 24 h or 48 h. **a** There are no change of the total number of bovine fibroblasts treated with IP for both 24 h and 48 h. **b**, **c** IP has no effect on the total number and the morphology of bovine colon epithelial cells for both 48 h. Scale bars = 250 μm. The means and standard deviations of triplicate determinations are presented

Bovine fibroblasts were used as tick feeding targets. Bovine colon epithelial cells were used as an example of the cell which can be affected by IP when it was administered in mammalian body as antimicrobial agent. After treatment with a high concentration of IP (50 μg/mL), no significant differences were found in the number of bovine fibroblasts and colon epithelial cells (Fig. 5a and b). Furthermore, there is no morphological change of bovine colon epithelial cells (Fig. 5c).

**Fig. 6:**
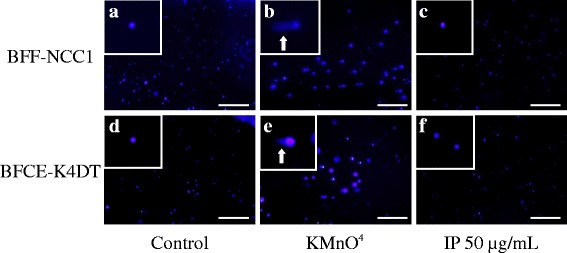
Neutral comet assay images of BFFs-NCC1 and BFCEs-K4DT cells after treatment with IP. **a**, **d** Untreated control cells show no damage to DNA. **b**, **e** Positive control cells treated with 25 μM KMnO4 show clear comet tails (arrows). **c**, **f** Each cell treated with IP (50 μg/ml) shows no damage to DNA as untreated control cells. Each inset has magnified views. Scale bars = 400 μm

The comet assay is a simple and sensitive method for studying DNA damage. In this neutral comet assay, each untreated control cell showed no comet tails (Fig. 6a and d), while positive control cells treated with KMnO4 showed some comet tails (Fig. 6b and e). Each cell treated with IP showed no comet tails similar to untreated control cells (Fig. 6c and f).

**Fig. 7:**
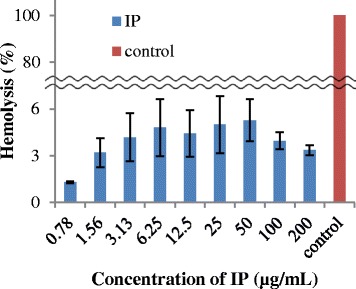
Hemolytic effect of IP. The hemolytic activity of IP was determined using human erythrocytes. In comparison with Triton X-100 treated erythrocytes as control cells (*red*), IP has no significant hemolytic activity against the cells even in 200 μg/ml. The means and standard deviations of triplicate determinations are presented

On the other hand, human erythrocytes were barely hemolyzed under the influence of IP: no significant hemolytic activity (<5 %) was observed after treatment of human erythrocytes with 200 μg/mL IP (Fig. 7). Similar patterns were found for other tick AMPs (IR, HAE, and OMBAC) as well (data not shown).

## Discussion

Arthropods such as ticks protect themselves by innate immunity, which involves antimicrobial peptides [34]. Ticks encounter diverse pathogens and therefore produce numerous antimicrobial factors [35]. With the increasing number of pathogens becoming resistant to conventional antibiotics, tick AMPs may serve as templates for the development of novel anti-infective agents. Yet, there have been several reports about antimicrobial activity of the tick AMPs [9, 11–13, 16]. IP from *I. persulcatus* is a potential candidate for a novel anti-infective agent because of its antimicrobial activity against gram-positive bacteria [13, 18].

IP belongs to the defensin family and contains six disulfide-paired cysteines. The structure of IP was found to be more stable with an S-S bond than that without an S-S bond by homology modeling and molecular dynamics. Therefore, we used an IP structure with an S-S bond in other experiments. Several groups have shown that analogues of invertebrate and vertebrate AMPs belong to the defensin family that lacked the S-S bond retained their broad-spectrum activity [36–38]. However, IP with a three-dimensional structure was more effective against gram-positive bacteria compared to that with a primary structure [18]. Therefore, the three-dimensional structure of IP with an S-S bond may be important for *I. persulcatus* to protect itself from infective bacteria. The structure of IP with an S-S bond is a distinctive arthropod structure containing one α helix and two β sheets, and has cationic property because of the positive charge caused by Arg and Lys residues. Almost all AMPs are cationic and amphipathic [34], because this characteristic of AMPs is related to their antimicrobial activity. Many AMPs approach microbes with their cationic parts, because microbial membranes are rich in anionic phospholipids, and cause pore formation with their amphipathic structure [39]. It is believed to be very difficult for bacteria to develop resistance to AMPs because most AMPs kill bacterial cells quickly by their actions on the entire bacterial cytoplasmic membrane or through other complex mechanisms [40, 41]. IP is also cationic and amphipathic (Fig. 3), so its mechanism of action may be similar to that of other AMPs, suggesting that it may be difficult for bacteria to develop resistance to IP. It has been reported that the antibacterial mechanism of tick AMP can disrupt the bacterial membrane [17].

We found that IP inhibited the growth of all 9 clinical isolates of MSSA and MRSA at MIC of 1.25 μg/mL and 2.5 μg/mL, respectively (Table 3). Between MSSA and MRSA, MIC of IP was not different. BMAP28, which belongs to the cathelicidin family of AMP, was more effective against MSSA than against MRSA [31, 42]. BMAP28 and tick AMPs both cause physical membrane disruption, but do not belong to the same family. BMAP28 has a different structure compared to that of IP, for example: it does not have an S-S bond or β sheet. These structural differences may underlie the differences in antimicrobial activity against normal and drug-resistant bacteria. Comparing the antimicrobial activity of each tick AMP against MRSA, we observed a large difference in MICs among tick AMPs. This result suggests that although tick AMPs have similar characteristics, such as large number of cysteines and S-S bond, antimicrobial activity was affected by changes in amino acid sequences and properties. Excluding HAE, antimicrobial activity of tick AMPs against MRSA may be related to the hydrophobicity of amino acid sequence. In the short time killing assay, IP was more effective against MRSA than against MSSA (Fig. 4). Moreover, IP showed dose-dependent bactericidal activity against both MSSA and MRSA. This anti-staphylococcal activity of IP was related so that *S. aureus* was not isolated from *I. persulcatus* during feeding [16].

Host cytotoxicity of AMPs is a major limitation in their application as antimicrobial drugs [43]. Therefore, we investigated the effect of AMPs on bovine cell growth, morphology, and DNA damage and hemolytic activity against human erythrocytes. In the cell growth experiment, IP did not affect bovine fibroblasts and colon epithelial cells growth, and morphology (Fig. 5). Not only that, but DNA damage was not detected in each cell treated with IP in neutral comet assay (Fig. 6). This result suggests that IP did not impair cell function. Moreover, in the hemolytic assay, we observed minimal hemolysis (<6 %) with IP, even at high concentrations (Fig. 7). AMPs, such as the honeybee AMP melittin, which is a linear cationic peptide without cysteines and has antimicrobial activity against MRSA, show hemolytic activity [44, 45]. Therefore, IP was an AMP without hemolytic activity. Taken together, IP cannot be toxic towards fibroblasts, colon epithelial cells and erythrocytes, which could overcome a challenge in their development as pharmaceutical drugs.

Treating MRSA infection is challenging owing to the remarkable ability of *S. aureus* to develop resistance to multiple antibiotics, thus limiting the number of viable therapeutic options [46, 47]. New anti-MRSA drugs called DAP were developed recently, but DAP-resistant bacteria have already been reported [48]. Therefore, there is an urgent need to develop novel antimicrobials with unique mechanisms of action to combat MRSA, one of which is AMP [4]. It has been reported that tick AMP derived from *Ornithodoros moubata*, with an amino acid sequence similar to that of OMBAC (91 %) (Table 2), had a low MIC against MRSA [17]. In this study, compared to other tick AMPs and mammalian AMP, IP had strongest antimicrobial activity against MRSA (Table 4). Furthermore, IP is non-toxic to human cells. Hence, IP may be a better candidate for a new anti-MRSA therapy.

## Conclusions

In this study, we predicted the structure of persulcatusin (IP), which is an AMP derived from the tick that causes Lyme disease. The structural integrity of IP is maintained by the S-S bond, unlike AMPs from the cathelicidin family such as BMAP28. IP is both cationic and amphipathic, and this characteristic and structural feature might be related to its antimicrobial activity. Moreover, IP showed antimicrobial activity against *S. aureus* but not toxic against mammalian and human cells such as fibroblasts, colon epithelial cells and erythrocytes. In particular, we found that IP has strong antimicrobial activity against MRSA.

## Abbreviations

AMP: antimicrobial peptide
IP: persulcatusin
MSSA: methicillin-susceptible *Staphylococcus aureus*
MRSA: methicillin-resistant *S. aureus*
MIC: minimum inhibitory concentration
RMSD: root mean square deviation
TS: trypto soya
